# Fast dissolving strips: A novel approach for the delivery of verapamil

**DOI:** 10.4103/0975-7406.72133

**Published:** 2010

**Authors:** S. Kunte, P. Tandale

**Affiliations:** Department of Pharmaceutics School of Pharmacy and Technology Management, NMIMS, Vile Parle (West), Mumbai, India

**Keywords:** Fast dissolving strips, HPMC E6, maltodextrin, verapamil

## Abstract

**Objective:**

Fast dissolving drug delivery system offers a solution for those patients having difficulty in swallowing tablets/capsules etc. Verapamil is a calcium channel blocker used as an antianginal, antiarrhythmic, and antihypertensive agent with extensive first pass metabolism which results in less bioavailability. This work investigated the possibility of developing verapamil fast dissolving strips allowing fast, reproducible drug dissolution in the oral cavity; thus bypassing first pass metabolism.

**Materials and methods:**

The fast dissolving strips were prepared by solvent casting technique with the help of HPMC E6 and maltodextrin. The strips were evaluated for drug content uniformity, film thickness, folding endurance, in vitro disintegration time, in vitro dissolution studies, surface pH study, and palatability study.

**Results:**

Official criteria for evaluation parameters were fulfilled by all formulations. Disintegration time showed by formulations was found to be in range of 20.4–28.6 sec. Based on the evaluation parameters, the formulation containing 2% HPMC E6 and 3.5% maltodextrin showed optimum performance against other formulations.

**Conclusion:**

It was concluded that the fast dissolving strips of verapamil can be made by solvent casting technique with enhanced dissolution rate, taste masking, and hence better patient compliance and effective therapy

Recently, fast dissolving drug delivery systems have started gaining popularity and acceptance as new drug delivery systems, because they are easy to administer and lead to better patient compliance. These delivery systems either dissolve or disintegrate in the mouth rapidly, without requiring any water to aid in swallowing.[1] They also impart unique product differentiation, thus enabling use as line extensions for existing commercial products. This novel drug delivery system can also be beneficial for meeting the current needs of the industry are improved solubility/stability, biological half life and bioavailability enhancement of drugs.[2,14]

Although oral disintegrating tablets have an advantage of administration without choking and fast disintegration; the disintegrated materials contained in them are insoluble and remain until swallowing. In such cases formulation of fast dissolving film will be advantageous.[3,16] Verapamil is used as voltage dependent calcium channel blocker. And it has been widely used in treatment of arrhythmia, angina and hypertension.[4] In the treatment of previously stated heart diseases, it acts by slowing down heart impulses through AV and SA node and causing dilatation of blood vessels. It is having high first pass metabolism which results in poor bioavailability (10–30%).[5] In view of substantial first pass effect and its shorter plasma half-life, is an ideal drug candidate for rapid release drug delivery system.

## Materials and Methods

### Materials

Verapamil hydrochloride was received as a gift sample from Lubrizol India Ltd., Mumbai. HPMC E6 (Colorcon Asia Ltd, India), maltodextrin (Research Lab Fine Chem Ind., Mumbai), glycerol (Qualigens fine chemicals, Mumbai), aspartame (Chagzhou Kelong Chem Ltd, China) were used as film base materials. All the chemicals used were of analytical grade.

### Preparation of fast dissolving strips

Hydroxypropylmethyl cellulose (HPMC) is known for its good strip-forming properties and has excellent acceptability.[6,9] Maltodextrin is classified as a complex carbohydrate, but acts like a simple carbohydrate in the body. It acts as film-forming agent, solubilizer, and imparts sweetness to the formulation.[7,8] For the fabrication of films, glycerol was used as a plasticizer and aspartame was used as a sweetener.[1]

The aqueous dispersion was prepared by dissolving HPMC E6, maltodextrin in distilled water maintained at 70°C [Table 1]. The suspension was used after 24 h to remove all the air bubbles entrapped. The active ingredient was added in the required quantity. Then remaining ingredients were added in the proportions given in Table 1. The solution was coated on teflon petri plate and then kept for drying at 75°C for first 30 min and then it was decreased to 45°C for next 24 h. The resultant film was cut into the dimension of 2 × 2 cm in size, in which 80 mg verapamil was included [Table 1].

**Table 1:** Formulation of fast dissolving strip of verapamil hydrochloride

Ingredients	F1 (%)	F2 (%)	F3 (%)	F4 (%)	F5 (%)
HPMC E6	2	2	2	5	2
Maltodextrin	1	2.5	3.5	2.5	-
Glycerol*	15	15	15	15	15
Aspartame*	7	7	7	7	7
Flavorant raspberry	0.05	0.05	0.05	0.05	0.05
Sodium benzoate	0.5	0.5	0.5	0.5	0.5
Colorant amaranth	0.05	0.05	0.05	0.05	0.05
Distilled water	qs	qs	qs	qs	qs

Quantities are expressed in terms of w/v.

* Quantities are expressed in terms of w/w of polymer

### Evaluation of fast dissolving strips

#### Uniformity of dosage units of the oral strips

**Figure 1:**
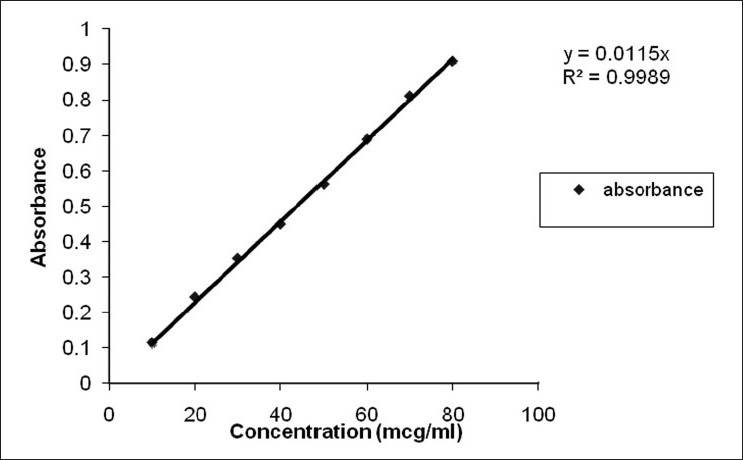
Standard curve of verapamil in phosphate buffer pH 6.6

**Figure 2:**
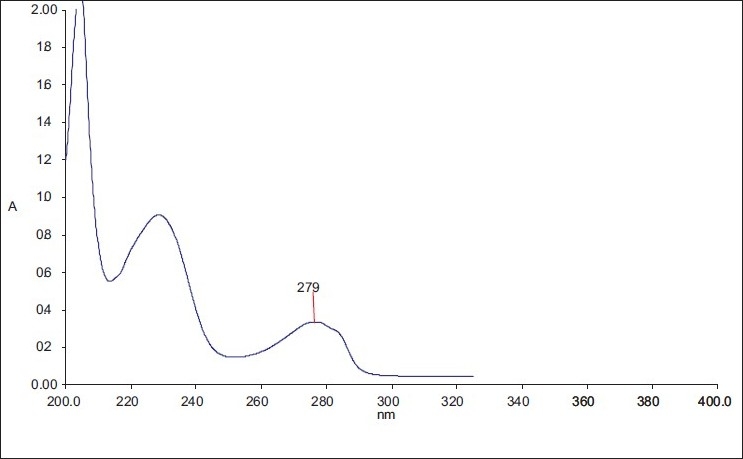
UV-spectrum of verapamil

The content uniformity of dosage units of the oral film preparation was tested for verapamil HCl using UV spectroscopy. According to the USP standards, the contents of preparations should lie between the limits 90% and 110%.[17] The results were expressed as mean of three determinations. The drug content was determined by using a standard calibration curve of verapamil [Figure 1]. The UV spectrum of verapamil is shown in [Figure 2].

#### Strip thickness measurement

The thickness of the fast dissolving film (2 × 2 cm) was measured using film thickness tester (Mitutoyo, Japan). The thickness of each strip was tested at three different positions.[1]

#### Folding endurance study

It was measured manually for the prepared fast dissolving film (2 × 2 cm). A strip was repeatedly folded at the same place till it broke. The number of times the film could be folded at the same place without breaking gave the value of folding endurance.[10]

#### In-vitro disintegration study

Disintegration test was performed in the USP disintegration time testing apparatus (Electrolab, Mumbai). Phosphate buffer (pH 6.6) was used as medium. The films were placed in the tubes of the container and disintegration time was recorded.[11]

#### In-vitro dissolution study

The dissolution test was performed according to the USP type II basket apparatus (Campbell Electronics, Mumbai). Test solution was 900 mL of phosphate buffer (pH 6.6) at 37±0.5°C with a rotation rate of 50 rpm. 10 ml aliquots of samples were taken at time intervals from 1 to 30 min and the same volume of fresh of phosphate buffer (pH 6.6) was replenished. Verapamil HCl concentrations were assayed spectrophotometrically at 278 nm (PerkinElmer Lambda 25, USA). The results were expressed as mean of three determinations.[11,12,15]

#### Surface pH study

The surface pH of fast dissolving strip was determined in order to investigate the possibility of any side effects *in vivo*. As an acidic or alkaline pH may cause irritation to the oral mucosa, it was determined to keep the surface pH as close to neutral as possible. A combined pH electrode was used for this purpose. Oral strip was slightly wet with the help of water. The pH was measured by bringing the electrode in contact with the surface of the oral film. The experiments were performed in triplicate, and average values were reported.

*Palatability study*. All the subjects were completely informed concerning the pertinent details and the purpose of the study. A written consent form was supplied, understood, and signed by each subject prior to dispensing the test materials. Films were randomly administered to healthy human volunteers between age group 20–40 years (*n* = 6; 4 males and 2 females) at 15 min time intervals. A specimen of 4 cm^2^ was placed in the oral cavity by the volunteer, directly on the tongue. All the subjects were asked to evaluate fast dissolving strips on the basis of three parameters: taste, after bitterness, and mouth feel.[13] A key for the same is given in Table 2.

**Table 2:** Key for the evaluation of palatability study

Parameter	Taste	After bitterness	Mouth feel
0	Not good	Not bitter	Not good
+	Good	Slightly bitter	Good
++	Very good	Bitter Very	good
+++	Excellent	Very bitter	Excellent

## Result and Discussion

All the strips were found to contain an almost uniform quantity of the drug, as per content uniformity studies [Table 3] indicating reproducibility of the technique. Drug content in the films was evaluated and the values were found to be between 99.36% and 100.78% for five different formulations. As per the USP requirements, the formulations found to meet the criteria for content uniformity.

**Table 3:** Evaluation of fast dissolving strip of verapamil hydrochloride

Formulation code	Drug content (%)	Film thickness (mm)	Folding endurance	Disintegration time (seconds)	Surface pH
F1	99.31±1.09	0.328±0.01	271.667±0.57	24.667±0.57	6.91±0.08
F2	100.78±0.5	0.2267±0.01	298.334±2.08	23±3.21	6.73±0.21
F3	100.49±1.63	0.206±0.005	300±1	20.334±1	6.913±0.08
F4	99.36±1.11	0.295±0.01	262.667±2.51	28.667±0.57	6.86±0.1
F5	100.17±0.26	0.24±0.01	257.334±1.52	27±1	6.85±0.07

Results are expressed in terms of mean ± SD (*n* = 3).

In this study, strip thickness was measured by using vernier calipers. As all the formulations contained different amounts of polymer, hence the thickess was varied in the range of 0.2–0.33 mm. With increase in maltodextrin concentarion from 1% to 3.5% reduction in thickness of the strip was observed [Table 3]. When concentration of HPMC E6 was increased from 2% to 5% in formulation F4, thickness of the strip increased.

Folding endurance was measured manually and it was found to improve with increase in concentration of maltodextrin from 1% w/w to 3.5% w/w. F10 batch containing higher amount of HPMC E6 scored less folding endurance as compared to earlier batches [Table 3].

*In-vitro disintegration study*. All the batches of fast dissolving strips for each formulation were found disintegrate in less than 30 sec.[1] *In vitro* disintegration time was found to decrease with increase in the amount of maltodextrin used in the formulations [Table 3]. F3 formulation found to gave minimum disintegration time (20.33±1) as compared to other preparations.

**Figure 3:**
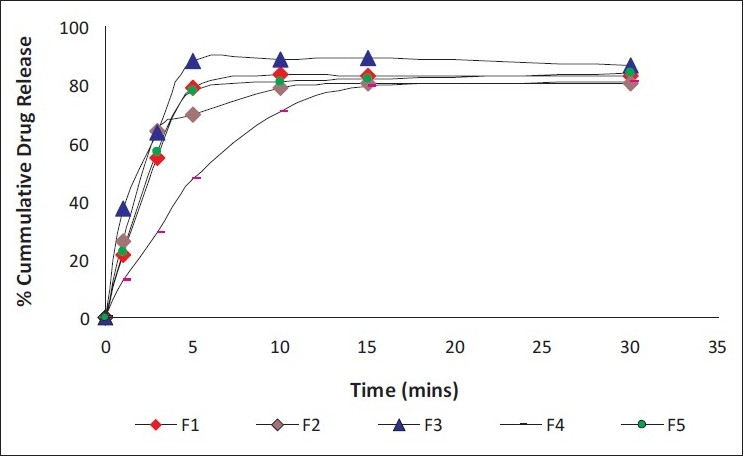
*In-vitro* drug release profile of formulations from F1 to F5

The *in vitro* drug release profile from the films of formulae F1 to F5 in phosphate buffer pH 6.6 is shown in Figure 3. Drug release rate was decreased with higher amount of HPMC E6 in formulation. After 15 minutes time interval more than 75% drug was released from batches. Thus batches meet the dissolution profile comparison criteria as defined in the Guidances such as USP30.[17]

The surface pH of the strips was ranging from 6.73± 0.21 and 6.91± 0.086. Since surface pH of films was found to be around neutral pH, there will not be any kind irritation to the mucosal lining of the oral cavity.

Strips of formulae F1 to F5 were evaluated for palatability on healthy human volunteers. The results of the in-vivo study are shown in Table 2 [key in Table 2]. With increase in the concentration of maltodextrin in the formulations from F1 to F3 mouth feel was improved [Table 4]. In F5 formulation after bitterness found and this was containing more HPMC E6 proportion as compared with maltodextrin. From these results, we can conclude that maltodextrin, which is the film-forming agent, also acts as a sweetener thus enhancing palatability of formulation [Table 4].

**Table 4:** Results of *in-vivo* performance of fast dissolving oral strips of batches F1 to F5 (*n* = 6)

Formulation Code	Palatability Study		
	Taste	After bitterness	Mouth feel
F1	+++	0	++
F2	++	+	+++
F3	+++	0	+++
F4	+	++	++
F5	++	+	++

## Conclusion

The study conclusively demonstrated significant taste masking of verapamil and rapid disintegration of fast dissolving oral strips. All the oral strips formulated with different concentrations of HPMC E6 and maltodextrin found to disintegrate in less than 30 seconds. Among the prepared formulations F3 showed minimum disintegration time of 20 seconds. *In vitro* and *in vivo* evaluation of the films confirmed their potential as an innovative dosage form to improve delivery of verapamil. Taste masked fast dissolving oral strips of verapamil are more palatable form without need of water during administration. Thus, the patient-friendly dosage form of bitter drugs such as verapamil can be successfully formulated using this technology and it can be especially useful for geriatric, bedridden, and noncooperative patients due to its ease of administration.

## References

[1] Dixit R, Puthli S (2009). Oral strip technology: Overview and future potential. J Control Release.

[2] Arya A, Chandra A, Sharma V, Pathak K (2010). Fast dissolving oral films: An innovative drug delivery system and dosage form. Int J ChemTech Res.

[3] Nishimura M, Matsuura K, Tsukioka T, Yamashita H, Inagaki N, Sugiyama T (2009). *In vitro* and *in vivo* characteristics of prochlorperazine oral disintegrating film. Int J Pharm.

[4] Drug card for verapamil. http://www.drugs.com/ppa/verapamilhydrochloride.html.

[5] Barends B, Vogelpoel H, Welink J, Amidon G, Junginger H, Midha K (2004). Biowaiver monographs for immediate release solid oral dosage forms based on biopharmaceutics classification system (BCS) literature data: Verapamil hydrochloride, propranolol hydrochloride, and atenolol. J Pharm Sci.

[6] Amin A, Mishra R (2009). Formulation development of taste-masked rapidly dissolving films of cetrizine hydrochloride. Pharm Technol.

[7] Cilurzo F, Cupone I, Minghetti P, Selmin F, Montanari L (2008). Fast dissolving films made of maltodextrins. Eur J Pharm Biopharm.

[8] Dzija M, Barkalow D, Chapdelaine A, Zyck D (2003). Edible film formulations containing maltodextrin US PATENT 2003/0035841.

[9] Rowe R (2003). Handbook of Pharmaceutical Excipients.

[10] Mahesh A, Shastri N, Sadanandam M (2010). Development of taste masked fast disintegrating films of levocetirizine dihydrochloride for oral use. Curr Drug Deliv.

[11] Patel R, Naik S, Patel J, Baria A (2009). Formulation development and evaluation of mouth melting film of ondansetron. Arch Pharm Sci Res.

[12] Mohammed A, Harish N, Charyulu R, Prabhu P (2009). Formulation of chitosan-based ciprofloxacin and diclofenac film for periodontitis therapy. Trop J Pharm Res.

[13] Dinge A, Nagarsenker M (2008). Formulation and evaluation of fast dissolving films for delivery of triclosan to the oral cavity. AAPS PharmSciTech.

[14] Mashru C, Sutariya V, Sankalia M, Parikh P (2005). Development and evaluation of fast-dissolving film of salbutamol sulphate. Drug Dev Ind Pharm.

[15] Siewert M, Dressman J, Brown CK, Shah VP (2003). FIP; AAPS. FIP/AAPS guidelines for dissolution/*in vitro* release testing of novel/special dosage forms. AAPS PharmSciTech.

[16] Shimoda H, Taniguchi K, Nishimura M, Matsuura K, Tsukioka T, Yamashita H (2009). Preparation of a fast dissolving oral thin film containing dexamethasone: A possible application to antiemesis during cancer chemotherapy. Eur J Pharm Biopharm.

[17] (2007). USP30-NF25, the United States Pharmacopoeia- National Formulary and its supplements.

